# Microbial symbionts and ecological divergence of Caribbean sponges: A new perspective on an ancient association

**DOI:** 10.1038/s41396-020-0625-3

**Published:** 2020-03-20

**Authors:** Christopher J. Freeman, Cole G. Easson, Kenan O. Matterson, Robert W. Thacker, David M. Baker, Valerie J. Paul

**Affiliations:** 1 Smithsonian Marine Station, Fort Pierce, FL USA; 2 Department of Biology, College of Charleston, Charleston, SC USA; 3 Halmos College of Natural Sciences and Oceanography, Nova Southeastern University, Dania Beach, FL USA; 4 Biology Department, Middle Tennessee State University, Murfreesboro, TN USA; 5 Department of Biology, University of Alabama at Birmingham, Birmingham, AL USA; 6 Smithsonian Institution, National Museum of Natural History, Washington, DC USA; 7 Department of Ecology and Evolution, Stony Brook University, Stony Brook, NY USA; 8 Smithsonian Tropical Research Institute, Box 0843-03092, Balboa, Republic of Panama; 9 The Swire Institute of Marine Science, School of Biological Sciences, University of Hong Kong, Hong Kong, PR China

**Keywords:** Microbial ecology, Stable isotope analysis

## Abstract

Marine sponges host diverse communities of microbial symbionts that expand the metabolic capabilities of their host, but the abundance and structure of these communities is highly variable across sponge species. Specificity in these interactions may fuel host niche partitioning on crowded coral reefs by allowing individual sponge species to exploit unique sources of carbon and nitrogen, but this hypothesis is yet to be tested. Given the presence of high sponge biomass and the coexistence of diverse sponge species, the Caribbean Sea provides a unique system in which to investigate this hypothesis. To test for ecological divergence among sympatric Caribbean sponges and investigate whether these trends are mediated by microbial symbionts, we measured stable isotope (*δ*^13^C and *δ*^15^N) ratios and characterized the microbial community structure of sponge species at sites within four regions spanning a 1700 km latitudinal gradient. There was a low (median of 8.2 %) overlap in the isotopic niches of sympatric species; in addition, host identity accounted for over 75% of the dissimilarity in both *δ*^13^C and *δ*^15^N values and microbiome community structure among individual samples within a site. There was also a strong phylogenetic signal in both *δ*^15^N values and microbial community diversity across host phylogeny, as well as a correlation between microbial community structure and variation in *δ*^13^C and *δ*^15^N values across samples. Together, this evidence supports a hypothesis of strong evolutionary selection for ecological divergence across sponge lineages and suggests that this divergence is at least partially mediated by associations with microbial symbionts.

## Introduction

Associations with microbial symbionts allow their hosts to exploit novel pools of nutrients and expand into ecological niches that would otherwise be inhospitable [1, 2]. The presence of reef-building corals on oligotrophic reefs that are the nutritional equivalent of “marine deserts” has long been a model for how these symbioses influence the ecological success of their animal hosts [3–5]. Nutritional symbioses are also widespread in other dominant organisms in reef ecosystems [4, 6, 7]. For instance, marine sponges are prolific filter feeders of live pico- and nanoplankton (2 μm or less) and detritus [8–10] on coral reefs, but they also host a microbial diversity that is unrivaled among other invertebrates [11, 12]. These microbial communities supply some sponge species with photosynthate [6, 13, 14], mediate the generation and recycling of nitrogen, a critical limiting nutrient on coral reefs [6, 9, 15–17], and facilitate the assimilation of dissolved sources of organic matter (DOM) [8–10, 18, 19].

The expansion of host metabolic capacity is likely a crucial feature of these interactions [20], but there is substantial variation in the abundance, diversity, and structure of these microbial communities across sponge species [21, 22]. In fact, pioneering work grouped sponges into one of two categories based on the overall abundance of their microbial communities (later referred to as high or low microbial abundance [HMA or LMA]) [23–25]. Fine scale analyses of sponge microbiomes, however, tend to paint a more nuanced picture of these communities. For example Easson and Thacker [26] found striking host specificity in microbiomes associated with 20 sponge species (both LMA and HMA) from the Caribbean coast of Panama, with even closely related species hosting significantly different microbiomes. The evidence for host specificity in these interactions has now been extended to include sponge species from other regions [27] and across a global scale [12], implying the presence of strong evolutionary selection for divergent microbiomes among sponge lineages.

Host sponge reliance on microbial metabolism also varies among sponge species. For instance, there is a continuum of host dependence on photosymbiont-derived carbon as some sponges host abundant and productive photosymbiont communities that supply their host with over 50% of its carbon (termed phototrophic sponges; *sensu* [28]) while other species lack these symbionts and rely on heterotrophic feeding to meet their energy demands [6, 14, 29–31]. In addition, while some symbiont communities are capable of producing new nitrogen via N_2_ fixation, oxidizing host-derived ammonium, or rapidly assimilating NO_3_^−^, sponge species that lack these symbiont groups are limited in their access to microbially mediated nitrogen transformations [6, 9, 15, 16, 24]. Finally, although dissolved organic carbon (DOC) can make up to 90% of the organic matter in seawater, sponge reliance on portions of three major pools of carbon (DOC, LPOC: living particulate organic carbon; and detritus) appears to be related to both microbial symbiont abundance and the physiology of each sponge species [8–10]. Previous work therefore highlights that sponge microbiomes facilitate the exploitation of novel resources, but that the specific resources acquired via these associations can depend on microbial abundance and host identity [1, 26].

Ecological theory predicts that high biodiversity within crowded ecosystems like coral reefs can be maintained (1) when competition among species is rare; for example, if resources do not limit populations or (2) when competition for limiting resources is prolific, leading to the exclusion of competitively inferior species and/or selection for adaptive traits that limit interspecific competition and promote coexistence [32, 33]. Sponges have expanded across ecological niches in marine ecosystems on a global scale, but they have been particularly successful on reefs within the Caribbean Sea, where there is high biomass, an average percent cover exceeding that of reef-building corals (15.9% [range of ~2–75%]), and high species diversity (>500 species) [34–36]. High biomass in the Caribbean was originally ascribed to elevated levels of organic carbon that favored heterotrophic feeding over sponge reliance on photosymbiont-derived nutrition [13, 29]; this proposed lack of carbon limitation in the Caribbean has been revisited and debated in recent years (see references within [37]). Despite the reported abundance of carbon resources, there is increasing evidence that microbial symbionts mediate resource use in Caribbean sponges by providing access to new sources of both carbon (DOC and photosynthate) and nitrogen (N_2_ fixation, assimilation of inorganic vs. organic, and recycling) [6, 10, 15, 16].

Although some studies have documented divergence in broad-scale resource use (measured as differences in the stable isotope ratios of carbon and nitrogen [*δ*^13^C and *δ*^15^N]) among Caribbean sponges that have variable associations with microbial symbionts, these studies are limited to specific locations or to only a few species, and have neither quantitatively tested for metabolic divergence across host phylogeny nor determined the relationship between microbial community structure and *δ*^13^C and *δ*^15^N values [24, 26, 31, 38–40]. Marine sponges are not amenable to classic experimental methods testing for competition and competitive exclusion [32, 41], and it would be difficult to isolate the influence that one sponge species has on another, coexisting species in situ. Thus, instead of testing for evidence of resource limitation or competition, our aim is to investigate evolutionary trends of ecological divergence across coexisting sponge species within the Caribbean. Based on the gaps in our understanding outlined above, our specific objectives are to (1) test for divergence in microbial community structure and *δ*^13^C and *δ*^15^N values of tissue from coexisting sponge species on individual reefs in the Caribbean; (2) investigate the stability of this divergence across large spatial scales in this ocean basin; (3) assess whether divergence in *δ*^13^C and *δ*^15^N values and microbiome diversity is linked to host phylogeny; and (4) investigate whether trends in *δ*^13^C and *δ*^15^N values across sponge samples mirror those for microbiome community structure or diversity.

## Materials and methods

### Sponge collection

Sponge species were collected from at least one site within four geographic regions spanning more than 15° of latitude (~1700 km) within the Caribbean Sea (Supplementary Tables S1 and S2; Supplementary Figs. S1, S2). Individual regions included the Bocas del Toro archipelago of Panama, the Miskito Cays of Honduras, the Mesoamerican barrier reef of Belize, and the Florida Keys (Supplementary Fig. S2). At each site, replicate small (3–5 ml) samples of dominant and conspicuous sponge species (Supplementary Table S1) were collected by SCUBA using a dive knife and placed into individual bags containing seawater for transport back to the laboratory. Sponge samples always included a cross section with both inner and outer tissue regions to standardize collections and sample across the entire body of the sponge. Collections frequently included eight of the ten most dominant Caribbean species [36] and species previously designated as both HMA and LMA sponges [24]. Samples were preserved, processed, and prepared for *δ*^13^C and *δ*^15^N analysis [31, 38]; see Supplementary Methods S1 for more details; Supplementary Table S3. Sponges were identified to species and, if necessary, identities were verified via tissue histology and spicule preparations. Replicate subsamples of each sponge species were also preserved in 95% EtOH in 5 ml cryovials and frozen at −20 °C for analyses of microbial community structure.

### Stable isotope and chlorophyll *a* analyses

Stable isotope values (*δ*^13^C and *δ*^15^N) of bulk sponge tissue serve as a time-integrated record of the sources of carbon and nitrogen assimilated by a holobiont (including activities of both sponge and microbial cells) and any fractionation associated with symbiont or host metabolism or nutrient recycling. Within an individual reef, *δ*^13^C and *δ*^15^N values of sponge tissue therefore act as a metabolic “fingerprint” that integrates the physiological, metabolic, and ecological differences present across individual sponges [24, 38, 40, 42]; see Supplementary Methods S2 for additional discussion of the utility of *δ*^13^C and *δ*^15^N for studying resource use in sponges. Bulk sponge tissue samples were analyzed in the Stable Isotope Ratio Mass Spectrometry Laboratory at the University of Hong Kong as in [38]. Mean (±SE) precision during analysis was 0.1 (0.001) ‰ and 0.2 (0.03) ‰ for *δ*^13^C and *δ*^15^N, respectively. Isotope values are expressed in delta (*δ*) notation in units per mille (‰). Values of the elemental composition (%C, %N, and C:N) of each sample of sponge tissue were also provided. Elemental values provide important information about how biomass-associated pools of carbon and nitrogen vary across sponge species and allowed us to test whether our trends in *δ*^13^C and *δ*^15^N values were strongly influenced by structural differences in sponge tissue. Photosymbiont abundance (as determined by chlorophyll *a* [chl *a*] concentration) was quantified in sponges from sites in Honduras, Panama, and the Florida Keys as in [31] and expressed as μg chl *a* [g dry sponge tissue]^−1^. *Scopalina ruetzleri* samples were not analyzed for chl *a* because they were too small to provide tissue for both isotope and chl *a* analyses.

### Analyses of microbiomes

We surveyed the microbiomes within 294 individuals of the 14 most dominant sponge species from our isotope surveys within the Caribbean (Supplementary Table S4). Sponge sampling was most comprehensive within sites in the Bocas del Toro archipelago of Panama (10–13 species within each site) and on Wonderland Reef in the Florida Keys (12 species). Sponges from sites within three regions (Belize and North and South sites in Honduras [see [38] and Supplementary Fig. S2 for map and description of sites]) were pooled to provide a regional assessment of microbiome structure across species. For additional details of sample preparation, processing, and bioinformatics for these analyses, please see Supplementary Methods S3. In short, polymerase chain reaction was performed on extracted total genomic DNA following the 16S Illumina Amplicon protocol of the Earth Microbiome project (earthmicrobiome.org) and with barcoded 16S rRNA primers (515F and 806R; [43, 44]); sequencing on an Illumina MiSeq resulted in paired-end 250 base pair amplicons. Bioinformatics processing was conducted in R using the DADA2 pipeline [45, 46] and taxonomic assignments of amplicon sequence variants (ASVs) were carried out using the Silva database release 128 [47]. Prior to analysis, singleton reads were removed and ASV abundance was transformed to relative abundance (See Supplemental Methods S3 for more information).

### Statistical analyses

Statistical analyses and visualizations used the R packages [46] *picante* [48], *vegan* [49], *RVAidMemoire* [50], and *ggplot2* [51]. To test for ecological divergence across coexisting sponges, we assessed isotopic dissimilarity by calculating the Euclidean distance [38] between samples. Dissimilarity in microbial community structure (presence/absence + relative abundance of taxa) was calculated using the Bray–Curtis dissimilarity index. From these dissimilarity matrices, we measured the influence of sponge species, collection site, and microbial abundance groups (HMA or LMA) on dissimilarity in isotope values and microbial community structure across samples using a permutational multivariate analysis of variance (PERMANOVA) with the *adonis* function in *vegan* [49]. We included HMA/LMA categories in this and additional analyses below because this dichotomy has been used extensively to group structurally and functionally similar sponge species. To form the null model for the PERMANOVA, we controlled for between-site variation of individual sponge species by restricting shuffling during permutations to within sites. For isotope analysis, a PERMANOVA was carried out at each of 12 sites where at least seven sponge species were collected (Supplementary Tables S2, S3). All pairwise PERMANOVA analyses included a false discovery rate correction for multiple comparisons [50]. The PERMANOVA analysis provided an estimate of the proportion of overall dissimilarity across samples (via *R*^2^ values) that was attributed to host identity, collection site, and microbial abundance.

Ecological divergence within a site was also calculated by using intraspecific dispersion in *δ*^13^C and *δ*^15^N values to measure and visualize the isotopic “niche” of each species and calculate the overlap of this isotopic “niche” with those of other sympatric species within that site [52, 53]. This was carried out for 14 common Caribbean sponge species and visualized at four sites (the most diverse site in each geographic region) in bivariate (*δ*^13^C and *δ*^15^N) plots with isotopic niches represented as standard ellipse area (SEA_c_) according to [52]. The mean pairwise isotopic niche overlap among these species was also measured at eight of the most diverse sites using methods from [53] and visualized on a heatmap (see Supplementary Methods S4 for additional details). Unlike geometric estimates of isotopic niche overlap that characterize the boundaries of niche space (e.g., [52, 54] these methods allow for a probabilistic estimate of pairwise, directional niche overlap based on a Bayesian framework [53].

The alpha diversity of the microbial community within each sample was calculated as the observed richness (S), Shannon index (H′), and inverse Simpson’s index (D) using the *vegan* package [49].

To investigate ecological divergence across host phylogeny, we tested for a phylogenetic signal in *δ*^13^C and *δ*^15^N values, chl *a* concentration, ASV richness (S), Shannon index (H′), inverse Simpson’s index (D), and elemental composition (%C, %N, and C:N). To do this, we used mean values of species that were well represented in sampling across sites within at least two regions of the Caribbean. *Monanchora arbuscula* was not included in analyses for S, H′, and D due to low microbiome sample size. Bayesian phylogeny of sponge species was constructed using sequences from the small ribosomal subunit (18S) and the large ribosomal subunit (28S) downloaded from Genbank to assess genetic relatedness using methods similar to previous research [12]; see Supplementary Methods S5 and Supplementary Table S5 for details. Phylogenetic signal was calculated using the phylosignal function in the R package *picante*. This analysis assesses whether more closely related organisms possess more similar traits; higher values for K (Blomberg’s K) indicate trait patterns that are strongly linked to the evolutionary history of the organisms and low K values indicate trait patterns shaped by stochastic changes over evolutionary time [55].

Mantel tests were used to identify correlations between dissimilarity patterns in microbial community structure (Bray–Curtis dissimilarity) and isotope values (Euclidean distance). To test for relationships between elemental composition (%C, %N, and C:N) and isotope values (*δ*^13^C and *δ*^15^N) of sponge tissue, as well as between the mean *δ*^13^C and *δ*^15^N values of sponge tissue and microbiome community richness and diversity, we used linear regressions. In addition, we used an analysis of variance (ANOVA) to test for differences in the *δ*^13^C and *δ*^15^N values of sponge tissue between HMA and LMA groups. Both of these analyses were carried out in JMP (Ver 14).

## Results

### Ecological divergence across sponge species: *δ*^13^C and *δ*^15^N and chlorophyll *a*

**Fig. 1:**
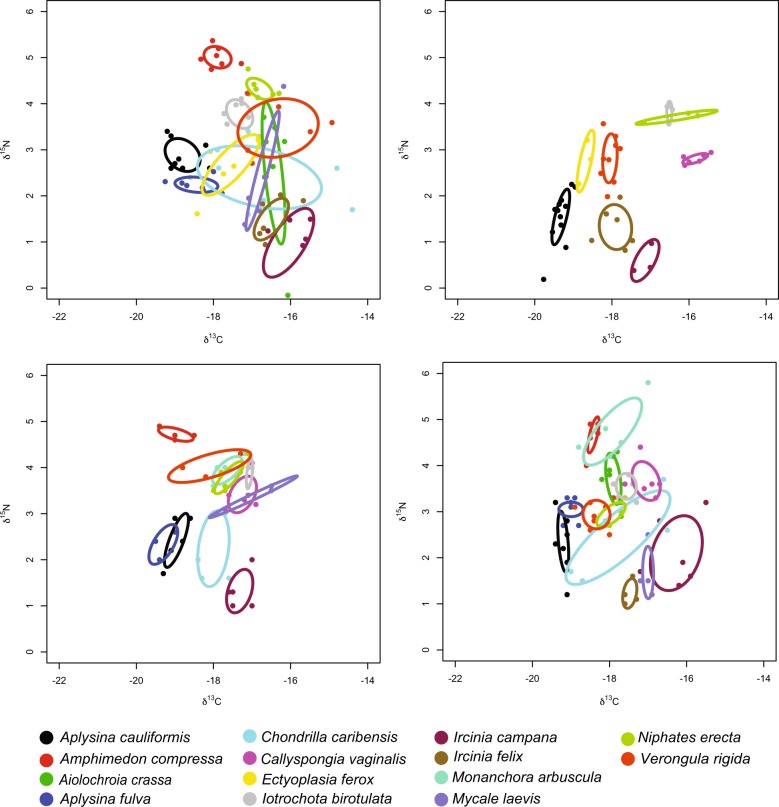
Bivariate (*δ*^15^N and *δ*^13^C) plots depicting the isotopic niches (as SEA_c_) of 14 common sponge species within the most diverse site within each of the four geographic regions of the Caribbean. Clockwise from upper left the sites are Wonderland Reef in the Florida Keys, South Water/Carrie Bow Channel on the Mesoamerican reef of Belize, Saigon Bay in Bocas del Toro, Panama, and Media Luna #2 from the Miskito Cays of Honduras. The figure from ML#2 was adapted from data in Freeman et al. [38]. Replicates of each species are represented by individual dots.

We collected individuals of 21 sponge species across 25 sites in the Caribbean that had variable species compositions (Supplementary Tables S1, S2, and S3 and Supplemental Figs S1, S2). Isotope values varied across sponge species, with host species identity accounting for ~59% of the dissimilarity in *δ*^13^C and *δ*^15^N values across samples from all sites within the Caribbean (PERMANOVA: df = 20, *F* = 61.01, *R*^2^ = 0.59, *p* = 0.001; Supplementary Table S6), and a range of 76–93% of the dissimilarity in *δ*^13^C and *δ*^15^N values within individual sites (Table 1). Collection site accounted for 16% of dissimilarity in isotope values across samples (PERMANOVA, df = 24, *F* = 39.05, *R*^2^ = 0.16, *P* = 0.001). Although *δ*^13^C and *δ*^15^N values varied between HMA and LMA groups, overall microbial abundance (HMA vs. LMA) accounted for only 20% of the dissimilarity among individual samples from across the Caribbean (PERMANOVA: df = 1, *F* = 219.49, *R*^2^ = 0.20, *p* < 0.001) and from 5 to 64% of the dissimilarity in *δ*^13^C and *δ*^15^N values at individual sites (Table 1). Isotopic niches (as shown as SEA_c_; [52] varied in their size due to differences in intraspecific dispersion of *δ*^13^C and *δ*^15^N, but, in general, isotopic niches were small within a site, leading to low overlap of sympatric sponges (Fig. 1). In fact, the average pairwise isotopic niche overlap among 14 common Caribbean sponges varied from 0 to 52%, with a median value of 8.2% (±SE 0.96%). Over 40% (75 out of 182) of these pairwise comparisons had a mean isotopic niche overlap of <5%, and 60% (108 out of 182) of tests had a mean isotopic niche overlap of <10% (Supplementary Fig. S3).

**Table 1:** *R*^2^ values (effect sizes) from permutational multivariate analysis of variance (PERMANOVA) showing the proportion of dissimilarity in stable isotope (*δ*^15^N and *δ*^13^C) values within a site explained by host species identity and overall microbial abundance (HMA or LMA).

Site (abbreviation)	Host species identity	Microbial abundance (HMA vs. LMA)
Saigon Bay (SB; BDT)	0.85***	0.16***
Crawl Cay (CC; BDT)	0.76***	0.27***
Isla Pastores (IP; BDT)	0.84***	0.33***
Caratasca #1 (C1; MC)	0.87***	0.48***
Media Luna #2 (ML2; MC)	0.87***	0.32***
Media Luna #3 (ML3; MC)	0.90***	0.26***
Glovers #1 (GS1; MR)	0.87***	0.05^ns^
Glovers #2 (GS2; MR)	0.85***	0.10^ns^
Raph’s Wall (RW; MR)	0.87***	0.15***
SW/CB Channel (SWCB; MR)	0.93***	0.38***
Tobacco Shallow (TS; MR)	0.92***	0.64***
Wonderland Reef (WR; FK)	0.76***	0.19***

Region of each site is denoted by: *BDT* Bocas del Toro, Panama, *MC* Miskito Cays, Honduras, *MR* Mesoamerican Reef, Belize, *FK* Florida Keys.

^ns^*P*  >  0.05, ***P  <  0.001 indicating a significant effect of host ID or microbial abundance on the isotopic differences between samples.

Species varied in their mean chl *a* concentration, with a range from over 450 μg chl *a* [g sponge tissue]^−1^ for *Ircinia campana* to 12 μg chl *a* [g sponge tissue]^−1^ for *Agelas conifera* (Supplementary Fig. S4). Seven species (all considered HMA) had high chl *a* values (>125 μg chl *a* [g sponge tissue]^−1^; [14]); the remaining low chl *a* species included both HMA and LMA species (Supplementary Fig. S4).

### Ecological divergence across sponge species: microbial community structure

The data for this study are available in the sequence read archive at NCBI (accession numbers: SAMN11832602–SAMN11833237; [56] BioProject number PRJNA544301). The 294 individuals of 14 sponge species collected as part of this study yielded 21,253 unique ASVs (17,539 after singleton reads were removed) that represented 80 microbial phyla according to the Silva taxonomic classification. Unique ASVs in a single sample ranged from 9 (in *C. vaginalis*) to 449 (in *M. laevis*). Only ten phyla had an average relative abundance of at least 1% (Supplementary Table S8).

**Fig. 2:**
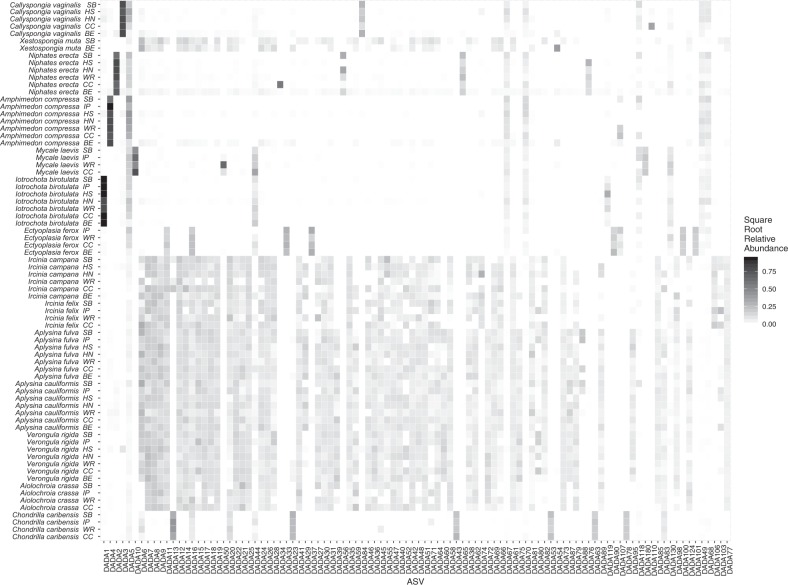
Mean relative abundance heatmap of the 100 most abundant microbial ASVs (organized from most to least abundant from left to right on *X*-axis) in each host species at each collection site or region within the Caribbean. These data are square-root transformed for ease of visualization. Site/region abbreviations are: SB Saigon Bay, IP Isla Pastores, HS Honduras South, HN Honduras North, WR Wonderland Reef, CC Crawl Cay, BE Belize.

There was a continuum of microbiome richness (median number of unique ASVs: range of 129 in *C. caribensis* to 305 in *A. cauliformis*) and diversity (Shannon index: range of 1.03 for *I. birotulata* to 4.72 for *A. cauliformis* and Inverse Simpson’s Index: range of 1.42 for *I. birotulata* to 65.36 for *A. cauliformis*) among these sponge species (Supplementary Table S7). The microbiomes of some sponge species were dominated by a single ASV that was absent within other species (Fig. 2). For instance, of the top 100 ASVs found in Caribbean sponges as part of this project, the microbiomes within *I. birotulata*, *C. vaginalis*, and *A. compressa* were dominated (81, 64, and 55%) by a single ASV (Fig. 2 and Supplementary Table S9).

We observed high host specificity in microbiome community structure (the presence and relative abundance of ASVs) at the level of the Caribbean, with 67% of the dissimilarity in microbial community structure across all samples explained by host identity (PERMANOVA: df = 13, *F* = 68.80, *R*^2^ = 0.67, *p* = 0.001). Collection site was significantly but weakly related to community structure (PERMANOVA, df = 6, *F* = 7.00, *R*^2^ = 0.03; *p* = 0.001), and host species and site exhibited a significant interaction (PERMANOVA, df = 54, *F* = 3.14, *R*^2^ = 0.13; *p* = 0.001). Microbial abundance classification (HMA or LMA) accounted for ~19% of the dissimilarity in microbial community structure across all samples (PERMANOVA, df = 1, *F* = 245.92, *R* = 0.19, *P* = 0.001). There was also a significant effect of host identity within individual sites or geographic regions, with a range of 79–88% (PERMANOVA) of the dissimilarity in microbial community structure across samples being driven by host identity (Table 2), and significant pairwise differences in microbial community structure between sympatric sponges (pairwise PERMANOVA; *P* ≤ 0.05 with FDR correction) except for *C. vaginalis* and *E. ferox* in Belize (*P* = 0.10) and *A. cauliformis* and *A. crassa* at Isla Pastores in Panama (*P* = 0.06). In contrast to host identity, microbial abundance (HMA vs. LMA) accounted for less of the dissimilarity in microbial community structure within sites (range of 19–36%; Table 2).

**Table 2:** *R*^2^ values (effect sizes) from permutational multivariate analysis of variance (PERMANOVA) showing the proportion of overall dissimilarity in microbial symbiont community structure within a site (top rows) or within a larger geographic region that includes multiple sites (bottom rows) explained by host species identity or overall microbial abundance (HMA or LMA).

Site (abbreviation; region; *N*)	Host species identity	Microbial abundance (HMA vs. LMA)
Sites		
Saigon Bay (SB; BDT; 13)	0.79***	0.23***
Crawl Cay (CC; BDT; 13)	0.81***	0.19***
Isla Pastores (IP; BDT; 10)	0.83***	0.19***
Wonderland Reef (WR; FK; 12)	0.86***	0.19***
Regions		
North Miskito Cays, Honduras (HN; MC; 8)	0.83***	0.36***
South Miskito Cays, Honduras (HS; MC; 8)	0.88***	0.35***
Belize (BE; MR; 10)	0.81***	0.20***

Region of each site is denoted by: *BDT* Bocas del Toro, Panama, *MC* Miskito Cays, Honduras, *MR* Mesoamerican Reef, Belize, *FK* Florida Keys, *N* number of species.

***P < 0.001.

### Ecological divergence across host phylogeny

**Fig. 3:**
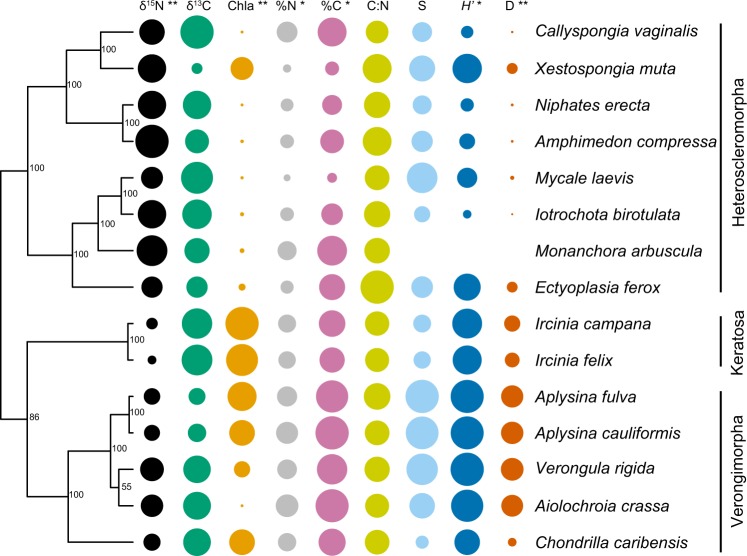
Phylogeny of Caribbean sponge species. To the right of the phylogeny, columns of circles represent the mean values of stable isotopes (*δ*^15^N and *δ*^13^C), chlorophyll a, elemental measurements (%N, %C, and C:N), and microbiome diversity (ASV richness, Shannon index, and inverse Simpson’s index) from all sites in the Caribbean where that species was collected. The size of the circle is proportional to the value of the particular metric. Taxonomic groups (to subclass) are shown for reference. The phylosignal function of the R package *picante* was used to test whether each trait displayed a significant phylogenetic signal. *P < 0.05, **P < 0.01.

There was a strong phylogenetic signal for δ^15^N across the Caribbean (*K* = 0.84, *P* = 0.001), with evidence of divergence in *δ*^15^N values across lineages of the subclasses Verongimorpha and Keratosa (depleted *δ*^15^N values) and species in the subclass Heteroscleromorpha (more enriched *δ*^15^N values) (Fig. 3). In contrast, variation in *δ*^13^C across sponge species was weakly linked with host phylogeny at the scale of the Caribbean (*K* = 0.38, *P* = 0.061; Fig. 3). Chl *a* values were tied to host phylogeny across the Caribbean (*K* = 0.75, *P* = 0.007), with generally elevated chl *a* values in members of the subclasses Verongimorpha and Keratosa and lower values in the subclass Heteroscleromorpha (Fig. 3). Elemental values (%C and %N) varied across host lineages (*K* = 0.41, *P* = 0.03 for %C and *K* = 0.44, *P* = 0.02 for %N), but there was only a weak phylogenetic signal for C:N (*K* = 0.40, *P* = 0.06; Fig. 3). There was evidence of divergence in microbiome richness across host phylogeny, but these trends were relatively weak (*K* = 0.40, *P* = 0.055) compared with those of microbiome diversity (measured via the Inverse Simpson’s index: *K* = 1.81, *P* = 0.001 and Shannon Index: *K* = 0.66, *P* = 0.011; Fig. 3).

### Link between ecological divergence and microbial community structure and diversity

Dissimilarity in microbial community structure was correlated with dissimilarity patterns in *δ*^15^N and *δ*^13^C values together (Mantel test: *r* = 0.20, *P* = 0.001), as well as with dissimilarity patterns in *δ*^15^N values (Mantel test: *r* = 0.24, *P* = 0.001) and, to a lesser extent, *δ*^13^C values (Mantel test: *r* = 0.04, *P* = 0.03). Mean *δ*^13^C and *δ*^15^N values of LMA sponges were higher than HMA sponges (ANOVA: df = 1, *F* = 106.9, *p* < 0.001 for *δ*^13^C and df = 1, *F* = 382.2, *p* < 0.001 for *δ*^15^N; Supplementary Fig. S5). Mean *δ*^15^N values were negatively related to microbiome diversity (mean Inverse Simpson’s Index; linear regression: *r*^2^ = 0.32, *p* = 0.03), but *δ*^15^N values were not influenced by microbiome richness (mean ASV richness; linear regression: *r*^2^ < 0.01, *p* > 0.05) (Supplementary Fig. S6). Mean *δ*^13^C values were not linked to microbiome diversity or richness (linear regression: *r*^2^ = 0.12, *p* = 0.22 and: *r*^2^ = 0.22, *p* = 0.09 for mean Inverse Simpson’s Index and mean ASV richness, respectively; Fig. S6).

The elemental composition of bulk sponge tissue varied across species, with a range from 9.2 [*M. laevis*] to 34.7 [*A. cauliformis*] for %C, from 2.4 [*M. laevis*] to 9.2 [*A. crassa*] for %N, and from 3.6 [*C. vaginalis*] to 5.4 [*E. ferox*]) for C:N (Supplementary Table S6 and Fig. 3). There were some significant, but weak relationships between the elemental composition of sponge tissue and *δ*^13^C and *δ*^15^N (Supplementary Fig. S7). Elemental values accounted for between 2 and 9% of the variation in *δ*^13^C and *δ*^15^N values across samples (Supplementary Fig. S7). There was also a strong positive relationship between elemental values, with the %C of sponge tissue explaining almost 90% of the variation in %N (Supplementary Fig. S7).

## Discussion

### Ecological divergence across individual sponge species

Our results show evidence of ecological divergence among coexisting sponge species on Caribbean reefs. On individual reefs and across the Caribbean, host sponge identity was the strongest determinant of dissimilarity in *δ*^15^N and *δ*^13^C values. In addition, although the isotopic niches (visualized as SEA_c_) of all species within a site were widespread across the *δ*^15^N and *δ*^13^C space, individual sponge species generally had narrow isotopic niches, with a median of <10% overlap between coexisting species [52, 53]. The segregation of common Caribbean sponge species across the niche axes represented by *δ*^15^N and *δ*^13^C values provides evidence of broad-scale partitioning of resources [38, 40]. Our findings, along with recent evidence of variation in host sponge reliance on nutrients from organic (both dissolved and particulate) and inorganic sources [6, 10, 24, 30, 57], are in agreement with ecological theory predicting increased selection, over evolutionary time, for adaptive traits that reduce competition through niche differentiation [32, 33]. In addition, with a stronger influence of host species identity than microbial abundance (HMA vs. LMA) (average across 12 sites of 88% for host species compared with 28% for microbial abundance), it is increasingly apparent [38, 40] that selective forces are driving ecological divergence at the level of host species within this ocean basin [58–60].

Coexisting species also had distinct microbiomes [12, 26], with a strong influence (range of 79–88 % within sites and 67% from across Caribbean) of host species identity on dissimilarity in microbiome community structure. In contrast, microbial abundance only accounted for between 19–36% of the variation within sites and 19% from across the Caribbean. Although microbial community structure was not strongly influenced by site (only 3% of total variance explained), more variation in microbiome community structure at larger spatial scales led to a reduction in the influence of host identity when tested across all of our sites. This variation is likely due to the presence of some site-specific microbes that could be commensals [12]. There was also a continuum of variation in microbiome diversity across these Caribbean sponge species, with the most striking trends in the values of the inverse Simpson’s Index. At one end of this continuum were species with a more even distribution of diverse microbial taxa (as in *V. rigida*, *A. crassa*, and *Aplysina* spp.), but at the other end of this continuum were *I. birotulata*, *C. vaginalis*, and *A. compressa*. In all three of these species, a single, unique ASV dominated the microbial community at all collection sites in the Caribbean. Striking host specificity in microbial community structure and diversity in Caribbean sponges [26] thus appears to be conserved across large spatial scales in this ocean basin.

### Divergence across host phylogeny

The evolutionary history of the host had a significant impact on broad-scale microbiome diversity (measured as the Inverse Simpson’s and Shannon Index), with more closely related species having more similar patterns of diversity than would be expected under a Brownian motion model of evolution (random walk). Although photosymbiont abundance was also linked to the phylogenetic history of the sponge host, this trait is not always a reliable proxy for host sponge reliance on photosynthate [6, 31]. Instead, reliance on photosymbiont-derived carbon is impacted by a combination of symbiont abundance, specificity, and productivity, and even closely related hosts have unique interactions with their photosymbiont communities [30, 31, 39]. The fact that *δ*^13^C values are not tightly constrained across host phylogeny may therefore be driven by variation in the dependence of sponge species on spatially and temporally variable sources of organic carbon (picoplankton, detritus, and DOC; [10, 37, 40, 61]).

The lack of a strong correlation between *δ*^13^C values and the phylogenetic history of host sponges makes the trends in *δ*^15^N values from these same sponge samples even more striking. Microbial symbionts mediate the nitrogen cycle within sponges, allowing transformations like N-fixation, nitrification, denitrification, and anaerobic ammonium oxidation [8, 17]. It is difficult to identify specific metabolic pathways that are driving divergence in the *δ*^15^N values across host phylogeny within this study, but higher *δ*^15^N values are generally associated with trophic enrichment from heterotrophic feeding and consistently depleted *δ*^15^N (−2 ‰ to ~0 ‰) values in some species (members of the genus *Ircinia* in the current study) are indicative of biological nitrogen fixation by diazotrophic bacteria [16]. Nitrification is likely a central function of some microbiomes [21, 62, 63], so nitrogen recycling within these symbioses may also be influencing *δ*^15^N trends across host species. For instance, nitrate release (as a proxy for nitrification) has been reported from *in situ* or laboratory-based experiments for some (*Aplysina* sp., *Ircinia* sp., *Aiolochroia crassa*, *Verongula rigida*, *Chondrilla caribensis*, and *Xestospongia muta*), but not all (not in *Callyspongia vaginalis*, *Niphates erecta*, and *Amphimedon compressa*) sponges from this study [15, 24, 64].

Our trends in *δ*^13^C and *δ*^15^N values across host phylogeny are especially interesting considering pioneering and recent work focused on organic carbon as the limiting nutrient on Caribbean reefs [29, 37, 65]. Microbiome richness, diversity, or structure may mediate carbon use by providing unique pathways for efficient carbon acquisition (DOC and photosynthate) that supplement host feeding on LPOC and detritus, but there is evidence of flexibility in carbon metabolism based on resource availability [37] and a lack of a relationship between microbiome composition and carbon flux [66]. This, along with data from our study suggest that there is relaxed selection pressure for physiological constraints in carbon metabolism across large spatial scales, and this may be influenced by high levels of carbon within the Caribbean [13, 29, 37]. Unlike carbon, nitrogen inputs into the Caribbean from rivers are thought to be generally low and productivity may therefore be nitrogen limited [61, 67]. Unique solutions to the challenge of nitrogen acquisition or processing across host lineages may therefore provide an adaptive advantage by reducing competition for this resource [66]. This, coupled with flexibility in carbon metabolism may also ensure that carbon skeletons are available for the production of biomolecules when nitrogen is available.

### Correlation between *δ*^15^N and *δ*^13^C values and microbiome community structure

*δ*^13^C and *δ*^15^N values differed between HMA and LMA groups, supporting the contention that sponges hosting abundant communities of microbial symbionts can more efficiently exploit and transform nutrients on these reefs [9, 10, 24]. Despite this pattern, within individual sites where coexisting sponge species have access to a similar pool of resources, microbial abundance accounted for a lower proportion of the overall dissimilarity in *δ*^13^C and *δ*^15^N values compared with host identity (microbial abundance: 28%, host identity: 88%). Therefore, it is unlikely that microbial biomass had a substantial influence on the isotope values of bulk sponge tissue. Instead, we identified a relationship between *δ*^15^N values and broad trends in microbiome diversity, with the inverse Simpson’s index explaining about 30% of the variation in mean *δ*^15^N values across host species. In addition, at an even finer scale, microbiome dissimilarity was strongly correlated with *δ*^15^N and *δ*^13^C dissimilarity across host sponges. Associations with specific symbionts can drive divergence in host resource use in terrestrial [68], deep-sea hydrothermal vent [69], and coral reef ecosystems [4, 70]. On coral reefs, ecological divergence at the level of host species has been reported in scleractinian corals, gorgonians, and sponges [4, 6, 29, 59], but these studies have focused mainly on carbon metabolism and fitness tradeoffs associated with adaptations that maximize light exposure and productivity. Interestingly, correlations between microbiome structure and trends in *δ*^15^N values across species were stronger than between *δ*^13^C alone or *δ*^15^N and *δ*^13^C together, supporting trends from our phylogenetic signal analysis that show divergence in nitrogen metabolism due to associations with microbial symbionts. Thus, although *δ*^15^N values of bulk sponge tissue are at least partially influenced by microbial biomass, our data also provide evidence that different sponge lineages are obtaining nitrogen in fundamentally different ways and that this variation is coupled to microbiome community structure. These results act as an important reminder that carbon is not the only nutrient shaping sponge holobiont evolution, and as our understanding of the complex nutrient cycling (C, N, S, and P) within and across sponge species increases [20], it is likely that additional life history patterns will emerge.

The values of *δ*^13^C and, to a lesser extent, *δ*^15^N can be influenced by variation in the biochemical (lipids, proteins, and carbohydrates) or structural (overall density and the proportion of skeletal elements like collagen fibers and spicules) composition of tissue [71]. Although the composition of sponge tissue (%C, %N, and C:N) varied across individual host species and also host phylogeny, there were only weak relationships between biomass-associated pools of elements (%C, %N, and C:N values) and *δ*^13^C and *δ*^15^N values (all less than 10% of variance). Thus, it is unlikely that the interspecific trends we observe in our *δ*^15^N and *δ*^13^C values are being strongly influenced by variation in the composition of sponge tissue [71–74].

## Conclusion

The high biomass and successful coexistence of diverse sponge species with different life history traits (morphologies, associations with microbial symbionts, and feeding strategies) has been cited as evidence of a lack of resource limitation on Caribbean reefs, as no single sponge type has gained a competitive advantage [13, 29, 37, 65]. Although there is no apparent evidence of competition leading to competitive exclusion in modern Caribbean sponge communities, sponge species in this ocean basin vary in the way that they exploit resources and this appears to be linked to microbial community structure [6, 10, 26, 60, 66, 75]. This ecological divergence and the conserved structural and functional traits reported across sponge species in this study may certainly have been shaped by past competition for limiting resources [32, 33, 58], but we recognize that processes besides competition could also drive these trends. For instance, because energetic and physiological constraints prevent individual sponge species from efficiently utilizing all available resources on a coral reef, different species might optimize their utilization of a particular resource over evolutionary time. In this case, ecological divergence and specialization in microbiome community structure and resource use could be the result of fitness tradeoffs associated with host sponge traits, feeding strategies, and members of the sponge microbiome. Indeed, mounting evidence suggests that sponge–microbe interactions have been shaped both by the evolutionary legacy of their hosts and current species-specific selective pressures to maintain these interactions. It is therefore likely that the metabolic divergence we observed is more strongly influenced by selective forces such as competition than by stochastic processes or random invasions of commensal microbes. However, these hypotheses should be investigated in future research.

Based on our observations, we posit that, over evolutionary time, the acquisition of novel symbiont taxa (or perhaps even shifts in the abundance of specific taxa shared across particular sponge species) allowed Caribbean sponges to exploit novel resources and expand into available niche space afforded by a combination of diverse sources of organic carbon and reduced competition with reef-building corals compared with other ocean basins [13, 61]. This expansion likely contributed to speciation and the colonization of diverse habitats in the Caribbean, leading to the formation of morphologically and trophically complex sponge communities [76]. In order to determine how resource use is influenced by both host and symbiont metabolism and test for fitness tradeoffs between divergent metabolic strategies across host phylogeny [4], future work should be carried out in disparate locations [9, 13, 29] using a standardized, integrative, and high-resolution approach [6, 9].

## Supplementary information

ASV Abundance

Supplementary Material
