# Supplementary material for: Microbial symbionts and ecological divergence of Caribbean sponges: A new perspective on an ancient association
Source: ISME J. 2020 Mar 20;14(6):1571–83. doi: 10.1038/s41396-020-0625-3 (PMC7242429; doi:10.1038/s41396-020-0625-3)
Supplement: Supplementary file 2 — Supplementary Material [file 41396_2020_625_MOESM2_ESM.docx]

**Supplementary Information for:**

**Microbial symbionts and ecological divergence of Caribbean sponges: A new perspective on an ancient association**

**Christopher J. Freeman^1,2^, Cole G. Easson^3,4^, Kenan O. Matterson^5,6^, Robert W. Thacker^7,8^, David M. Baker^9^, and Valerie J. Paul^1^**

**^1^Smithsonian Marine Station, Fort Pierce Florida, USA; freemanc@si.edu; paul@si.edu**

**^2^Department of Biology, College of Charleston, Charleston, South Carolina, USA**

**^3^Halmos College of Natural Sciences and Oceanography, Nova Southeastern University, Dania Beach, Florida, USA;** ceasson@nova.edu

**^4^Biology Department, Middle Tennessee State University, Murfreesboro, Tennessee, USA; cole.easson@mtsu.edu**

**^5^Department of Biology, University of Alabama at Birmingham, Birmingham, Alabama, USA;** [**kenanm@gmail.com**](mailto:kenanm@gmail.com)

**^6^Smithsonian Institution, National Museum of Natural History, Washington, District of Columbia, USA**

**^7^Department of Ecology and Evolution, Stony Brook University, Stony Brook, New York, USA;** [**robert.thacker@stonybrook.edu**](mailto:robert.thacker@stonybrook.edu)

**^8^Smithsonian Tropical Research Institute, Box 0843-03092, Balboa, Republic of Panama**

**^9^The Swire Institute of Marine Science, School of Biological Sciences, University of Hong Kong, Hong Kong, PR China;** [**dmbaker@hku.hk**](mailto:dmbaker@hku.hk)

**Corresponding author: Christopher Freeman; Department of Biology, College of Charleston, 66 George Street, Charleston, SC 29424; 817-707-5840; freemancj@cofc.edu**

**Contents:**

1. **Supplementary Methods**
2. **Supplementary Tables**
3. **Supplementary Figures**

**SUPPLEMENTARY METHODS**

**Supplementary Methods 1, sample preservation and preparation for isotope analysis:**

Due to logistical constraints at field stations or on research cruises (in Honduras), sponge samples for isotope analyses were either frozen at -20 °C (Florida Keys and Panama), dried at 60 °C for 24 h in a drying oven (Belize) or dried at 60 °C for 36 h using a Nesco (Two Rivers, WI, USA) FD-75A 700 W food dehydrator (Honduras; Freeman et al. 2014) in the field prior to transport back to the Smithsonian Marine Station (SMS). Isotope samples from the Florida Keys and Panama were lyophilized at the SMS and all dried bulk (including both host and microbial symbiont biomass) tissue samples were ground to a fine powder using a mortar and pestle. These drying methods are commonly used to prepare marine organisms for stable isotope analysis and comparisons of these methods indicate minimal shifts in *δ*^13^C and *δ*^15^N values (1 and references therein). Sponge tissue was exposed to 12 N HCl fumes for 12 h to remove carbonate and held at 60 °C for 24 h to remove residual acid. Homogenized sponge tissue was weighed into tared silver capsules to the nearest 0.001 mg for δ^13^C and δ^15^N analysis (2).

**Supplementary Methods 2, review of stable isotope analysis and analytical advances:**

As a holobiont containing both microbial and animal cell biomass, marine sponges have access to a myriad of nutrient sources: dissolved inorganic carbon (CO_2_, HCO_3_^-^) and nitrogen (N_2_, NH_4_^+^, NO_2_^-^ and NO_3_^-^), and particulate (picoplankton including phytoplankton, bacteria, viruses, and detritus; 3–5) and dissolved (both refractory compounds like humic acid derivatives and more labile components like sugars and other carbohydrates, proteins, vitamins, and hormones); (6,7) organic matter. The *δ*^13^C and *δ*^15^N values of bulk sponge tissue may thus reflect both host (sponge cells and structural materials like fiber skeleton and spicules that are produced by sponge cells) and symbiont (microbial cells) biomass and therefore provide a metric for metabolism that includes both members of the symbiosis.

The relative position of a sponge species within the two-dimensional (*δ*^13^C and *δ*^15^N) isotopic space provided by diverse nutrient sources within an individual reef can be used as a coarse metric for assessing ecological divergence (variation in resource use and processing) among coexisting species (8,9). In addition, comparisons are strengthened by recent analytical methods allowing for quantitative estimates of the size and placement of an organism’s “isotopic niche” relative to other sympatric species (8,10).

**Supplementary Methods 3, sample processing and bioinformatics for microbial community structure analysis:**

Seawater samples (500 ml) were collected at each site and filtered through 0.2 µm membranes at low pressure to collect samples of ambient microbes. Total genomic DNA was extracted from cross sections of sponge tissue (~0.25 grams) and ½ of seawater filter membranes using Qiagen PowerSoil Powerlyzer extraction kit and following the manufacturers protocol. After DNA extraction, polymerase chain reaction (PCR) was performed following the 16S Illumina Amplicon protocol of the Earth Microbiome project (earthmicrobiome.org) with barcoded 16S rRNA primers (515F and 806R; (11,12) and PCR products were cleaned using AMPure beads (Beckman Coulter). DNA concentration in cleaned products was measured using a Qubit fluorometer (Qubit). The concentration of each sample was diluted to 4 nM and then all samples were pooled in equal volumes. Pooled samples were then sequenced on an Illumina MiSeq platform following standard Illumina protocols for sample preparation and loading except that custom sequencing primers from the EMP protocol were used. A 500 cycle V2 chemistry MiSeq kit was used during sequencing, which yielded paired-end 250 base pair (bp) amplicons.

Bioinformatics processing was conducted in R using the DADA2 pipeline (13,14). Sequences were first trimmed to remove ambiguous bases (max N = 0), as well as sequences longer than 250 bp or shorter than 160 bp. Error rates were calculated using the default parametric error model in DADA2. Sequences were then dereplicated to infer sequence variants, paired-ends were merged, chimeras were removed, and a sequence table containing amplicon sequence variants (ASVs) was constructed. The Silva database (15) was used for taxonomic assignments of ASVs. A heatmap showing the 100 most abundant taxa in the dataset was generated using the R packages ggplot2 (16), reshape2 (17) and dplyr (18). Mean relative abundance of these microbial taxa was calculated for each species at each collection site. Sponge species were grouped based on phylogenetic relatedness, and relative abundance was square-root transformed before plotting

**Supplementary Methods 4, Isotopic Niche Overlap Methods:**

Species for these analyses were chosen based on their presence at three or more of these eight sites, with the highest number of species at a single site being 15 (Saigon Bay, Panama); *Xestospongia muta* was excluded because it was only collected at two of these eight sites. The pairwise overlap of sponge species was calculated at each of the eight sites, and these data were averaged to obtain mean directional estimations of within-site niche overlap between 14 pairs of sponge species across the Caribbean. Because all 14 species were not found at each site, the final mean pairwise estimations represent the directional overlap at only the sites where the two species co-occur. Mean overlap was calculated with values from at least three sites, with the exception of the species *Monanchora arbuscula* (Marb) and *Ircinia felix* (Ifel) that only co-occurred at two sites.

**Supplementary Methods 5, Phylogenetic Signal Data Analysis:**

A phylogeny of focal sponges was constructed using gene sequences previously deposited in GenBank (Table S2). Sequences corresponding to the small ribosomal subunit (18S) and the large ribosomal subunit (28S) were aligned separately using MAFFT 7.017 (19) and concatenated in the program Geneious (version 7.1.9). A Bayesian phylogeny was constructed using the MrBayes (version 3.2.1) software (20) as implemented by the computational resources of CIPRES (21). Two partitions were specified (18S, 28S), and for each partition, separate general time reversible models of evolution that incorporated a gamma distribution of substitution rates among sites and a proportion of invariant sites (GTR+I+G) were estimated (22). An independent gamma rate relaxed clock model was implemented with a birth-death process. Two taxa in the class Homoscleromorpha (*Plakortis halichondroide*s and *Plakina trilopha*) were constrained as outgroups. Three parallel runs consisted of 10 million generations for each run using four Markov chains and sampling every 100 generations. A consensus phylogeny was constructed from the three runs using a 25% burn-in. Phylogenetic signal in continuous traits was calculated using the phylosignal function in the R package *picante* (23), which tested whether more similar values of traits were associated with more closely related hosts more often than expected by chance.

Supporting References:

1. Bessey C, Vanderklift MA. Drying method has no substantial effect on δ15N or δ13C values of muscle tissue from teleost fishes. Rapid Commun Mass Spectrom. 2014;28(3):265–73.

2. Freeman CJ, Thacker RW. Complex interactions between marine sponges and their symbiotic microbial communities. Limnol Oceanogr. 2011;56(5):1577–86.

3. Maldonado M, Ribes M, van Duyl FC. Nutrient fluxes through sponges: Biology, budgets, and ecological implications. Adv Mar Biol. 2012;62:113–82.

4. McMurray SE, Stubler AD, Erwin PM, Finelli CM, Pawlik JR. A test of the sponge-loop hypothesis for emergent Caribbean reef sponges. Mar Ecol Prog Ser. 2018;588:1–14.

5. McMurray SE, Johnson ZI, Hunt DE, Pawlik JR, Finelli CM. Selective feeding by the giant barrel sponge enhances foraging efficiency. Limnol Oceanogr. 2016;61(4):1271–86.

6. Pawlik JR, Burkepile DE, Thurber RV. A vicious circle? Altered carbon and nutrient cycling may explain the low resilience of Caribbean coral reefs. Bioscience. 2016;66(6):470–6.

7. Fiore CL, Freeman CJ, Kujawinski EB. Sponge exhalent seawater contains a unique chemical profile of dissolved organic matter. PeerJ. 2017;5:e2870.

8. Layman CA, Araujo MS, Boucek R, Hammerschlag-peyer CM, Harrison E. Applying stable isotopes to examine food-web structure: an overview of analytical tools. Biol Rev. 2012;87:542–62.

9. Newsomes SD, Martinez C, Bearhop S, Phillips DL. A niche for isotopic ecology. Front Ecol Evol. 2007;5(8):429–36.

10. Swanson HK, Lysy M, Power M, Stasko AD, Johnson JD, Reist JD. A new probabilistic method for quantifying n-dimensional ecological niches and niche overlap. Ecology. 2015;96(2):318–24.

11. Caporaso JG, Kuczynski J, Stombaugh J, Bittinger K, Bushman FD, Costello EK, et al. QIIME allows analysis of high-throughput community sequencing data. Nat Methods. 2010;7:335.

12. Caporaso JG, Lauber CL, Walters WA, Berg-Lyons D, Huntley J, Fierer N, et al. Ultra-high-throughput microbial community analysis on the Illumina HiSeq and MiSeq platforms. ISME J. 2012;6(8):1621–4.

13. Callahan BJ, McMurdie PJ, Rosen MJ, Han AW, Johnson AJA, Holmes SP. DADA2: High-resolution sample inference from Illumina amplicon data. Nat Methods. 2016;13(7):581–3.

14. Team RC. R: A language and environment for statistical computing. Vienna, Austria: R Foundation for Statistical Computing; 2018.

15. Quast C, Pruesse E, Gerken J, Schweer T, Yilmaz P, Peplies J, et al. SILVA databases. In: Encyclopedia of Metagenomics: Genes, Genomes and Metagenomes: Basics, Methods, Databases and Tools. 2015. p. 626–35.

16. Wickham H. gglot2: elegant graphics for data analysis. New York: Springer-Verlag; 2016.

17. Wickham H. Reshaping Data with the reshape Package. J Stat Softw. 2007;21(12):1–20.

18. Wickham H, François R, Henry L, Müller K. dplyr: A Grammar of data manipulation. 2019.

19. Katoh K, Misawa K, Kuma K, Miyata T. MAFFT: a novel method for rapid multiple sequence alignment based on fast Fourier transform. Nucleic Acids Res. 2002;30(14):3059–66.

20. Ronquist F, Teslenko M, van der Mark P, Ayres DL, Darling A, Höhna S, et al. MrBayes 3.2: efficient Bayesian phylogenetic inference and model choice across a large model space. Syst Biol. 2012;61(3):539–42.

21. Miller MA, Pfeiffer W, Schwartz T. Creating the CIPRES Science Gateway for inference of large phylogenetic trees. In: 2010 Gateway Computing Environments Workshop (GCE). 2010. p. 1–8.

22. Huelsenbeck JP, Rannala B. Frequentist properties of Bayesian posterior probabilities of phylogenetic trees under simple and complex substitution models. Syst Biol. 2004;53(6):904–13.

23. Kembel SW, Cowan PD, Helmus MR, Cornwell WK, Morlon H, Ackerly DD, et al. Picante: R tools for integrating phylogenies and ecology. Bioinformatics. 2010;26(11):1463–4.

24. Erwin PM, Thacker RW. Incidence and identity of photosynthetic symbionts in Caribbean coral reef sponge assemblages. J Mar Biol Assoc United Kingdom. 2007;87(6):1683–92.

25. Weisz JB, Hentschel U, Lindquist N, Martens CS. Linking abundance and diversity of sponge-associated microbial communities to metabolic differences in host sponges. Mar Biol. 2007;152(2):475–83.

26. Gloeckner V, Wehrl M, Moitinho-Silva L, Gernert C, Schupp P, Pawlik JR, et al. The HMA-LMA dichotomy revisited: an electron microscopical survey of 56 sponge species. Biol Bull. 2014;227:78–88.

Supplementary Table S1: Sponge species collected from at least two sites within the Caribbean for stable isotope (δ^13^C and δ^15^N) analysis and their abbreviations used in some figures (the first letter of the genus name, followed by the first three letters of the species epithet).

| Sponge Species | Abbreviation |
| --- | --- |
| *Agelas conifera* (Schmidt, 1870) | Acon |
| *Agelas wiedenmayeri* Alcolado, 1984 | Awie |
| *Aiolochroia crassa* (Hyatt, 1875) | Acra |
| *Amphimedon compressa* Duchassaing & Michelotti, 1864 | Acom |
| *Aplysina cauliformis* (Carter, 1882) | Acau |
| *Aplysina fulva* (Pallas, 1766) | Aful |
| *Callyspongia (Callyspongia) fallax* Duchassaing & Michelotti, 1864 | Cfal |
| *Callyspongia (Cladochalina) plicifera* (Lamarck, 1814) | Cpli |
| *Callyspongia (Cladochalina) vaginalis* (Lamarck, 1814) | Cvag |
| *Chondrilla caribensis* Rützler, Duran, & Piantoni, 2007 | Ccar |
| *Desmapsamma anchorata* (Carter, 1882) | Danc |
| *Ectyoplasia ferox* (Duchassaing & Michelotti, 1864) | Efer |
| *Iotrochota birotulata* (Higgin, 1877) | Ibir |
| *Ircinia campana* (Lamarck, 1814) | Icam |
| *Ircinia felix* (Duchassaing & Michelotti, 1864) | Ifel |
| *Monanchora arbuscula* (Duchassaing & Michelotti, 1864) | Marb |
| *Mycale (Mycale) laevis* (Carter, 1882) | Mlae |
| *Niphates erecta* (Duchassaing & Michelotti, 1864) | Nere |
| *Scopalina ruetzleri* (Wiedenmayer, 1977) | Srue |
| *Verongula rigida* (Esper, 1794) | Vrig |
| *Xestospongia muta* (Schmidt, 1870) | Xmut |

Supplementary Table S2: Names (with site abbreviations and # of species collected), geographic region, depth range, and GPS coordinates of the 25 collections sites within the Caribbean.

| Site  (Abbreviation, # Species) | Region | Depth  Range | Latitude-N | Longitude-W |
| --- | --- | --- | --- | --- |
| Saigon Bay (SB, 15) | Bocas del Toro, Panama | 2-4 m | 9.346 | -82.258 |
| Crawl Cay (CC, 14) | Bocas del Toro, Panama | 4-5 m | 9.260 | -82.130 |
| Isla Pastores (IP, 13) | Bocas del Toro, Panama | 3-4 m | 9.243 | -82.344 |
| Becerros #1 (B1, 3) | Miskito Cays, Honduras | 3-4 m | 15.913 | -83.255 |
| Becerros #2 (B2, 4) | Miskito Cays, Honduras | 4-5 m | 15.951 | -83.272 |
| Caratasca #1 (C1, 8) | Miskito Cays, Honduras | 3-4 m | 16.024 | -83.316 |
| Caratasca #2 (C2, 3) | Miskito Cays, Honduras | 3-4 m | 16.030 | -83.319 |
| Media Luna #1 (ML1, 4) | Miskito Cays, Honduras | 2-3 m | 15.261 | -82.631 |
| Media Luna #2 (ML2, 15) | Miskito Cays, Honduras | 3-5 m | 15.186 | -82.618 |
| Media Luna #3 (ML3, 15) | Miskito Cays, Honduras | 3-5 m | 15.139 | -82.582 |
| Media Luna #4 (ML4, 5) | Miskito Cays, Honduras | 3-5 m | 15.122 | -82.587 |
| Vivorillos #1 (V1, 3) | Miskito Cays, Honduras | 2-5 m | 15.837 | -83.291 |
| Vivorillos #2 (V2, 6) | Miskito Cays, Honduras | 2-5 m | 15.863 | -83.306 |
| Deep Tobacco (DT, 6) | Mesoamerican reef, Belize | 15-18 m | 16.896 | -88.057 |
| Curlew (CW, 2) | Mesoamerican reef, Belize | 2-3 m | 16.789 | -88.078 |
| Glovers #1 (G1, 7) | Mesoamerican reef, Belize | 15-16 m | 16.751 | -87.788 |
| Glovers #2 (G2, 9) | Mesoamerican reef, Belize | 15-18 m | 16.736 | -87.813 |
| Glovers #3 (G3, 2) | Mesoamerican reef, Belize | 12-15 m | 16.713 | -87.865 |
| Glovers Atoll (GA, 6) | Mesoamerican reef, Belize | 2-3 m | 16.731 | -87.852 |
| Patch Reef (PR, 5) | Mesoamerican reef, Belize | 2-4 m | 16.778 | -88.114 |
| Raph’s Wall (RW, 11) | Mesoamerican reef, Belize | 12-15 m | 16.779 | -88.075 |
| Station (S, 3) | Mesoamerican reef, Belize | 8-10 m | 16.802 | -88.079 |
| SW/CB (SW/CB, 15) | Mesoamerican reef, Belize | 2-5 m | 16.811 | -88.079 |
| Tobacco Shallow (TS, 10) | Mesoamerican reef, Belize | 2-5 m | 16.889 | -88.065 |

| Wonderland (WR, 13) | Florida Keys | 6-8 m | 24.560 | -81.501 |
| --- | --- | --- | --- | --- |

Supplementary Table S3: Sites where each sponge species was collected (denoted by an “X”) within the Caribbean for isotope (δ^15^N and δ^13^C) values. The total number of each sponge species collected across all sites for isotope values is also shown for reference

| Site | *Agelas conifera* | *Agelas wiedenmayeri* | *Aiolochroia crassa* | *Amphimedon compressa* | *Aplysina cauliformis* | *Aplysina fulva* | *Callyspongia (Callyspongia) fallax* | *Callyspongia (Cladochalina) plicifera* | *Callyspongia (Cladochalina) vaginalis* | *Chondrilla caribensis* | *Desmapsamma anchorata* | *Ectyoplasia ferox* | *Iotrochota birotulata* | *Ircinia campana* | *Ircinia felix* | *Monanchora arbuscula* | *Mycale (Mycale) laevis* | *Niphates erecta* | *Scopalina ruetzleri* | *Verongula rigida* | *Xestospongia muta* |
| --- | --- | --- | --- | --- | --- | --- | --- | --- | --- | --- | --- | --- | --- | --- | --- | --- | --- | --- | --- | --- | --- |
| Total N Sampled (δ^15^N and δ^13^C) | 16 | 16 | 48 | 62 | 92 | 63 | 13 | 18 | 46 | 33 | 17 | 39 | 60 | 45 | 49 | 27 | 41 | 61 | 10 | 68 | 41 |
| Saigon Bay |  |  | X | X | X | X |  |  | X | X | X |  | X | X | X | X | X | X |  | X | X |
| Crawl Cay |  |  | X | X | X | X |  |  | X | X |  | X | X | X | X | X | X | X |  | X |  |
| Isla Pastores |  |  | X | X | X | X |  |  | X | X |  | X | X | X | X |  | X | X |  | X |  |
| Becerros #1 |  | X |  | X |  |  |  |  |  |  |  |  |  |  |  |  |  | X |  |  |  |
| Becerros #2 |  |  |  |  | X |  | X |  |  |  |  |  | X | X |  |  |  |  |  |  |  |
| Caratasca #1 |  |  |  |  | X | X | X |  | X |  |  |  | X | X |  |  |  | X |  | X |  |
| Caratasca #2 |  |  |  |  | X | X |  |  |  |  |  |  | X |  |  |  |  |  |  |  |  |
| Media Luna #1 |  |  |  | X | X | X |  |  |  |  |  |  |  |  |  |  |  |  |  | X |  |
| Media Luna #2 | X |  |  | X | X | X | X | X | X | X |  |  | X | X | X | X | X | X |  | X |  |
| Media Luna #3 | X |  | X | X | X |  | X | X | X |  |  | X | X | X |  | X | X | X |  | X |  |
| Media Luna #4 |  |  | X |  | X | X |  |  |  |  | X |  |  |  |  |  |  |  |  | X |  |
| Vivorillos #1 |  | X |  |  |  |  |  |  |  |  |  |  |  |  | X |  |  |  |  | X |  |
| Vivorillos #2 |  |  |  |  | X | X |  |  | X |  |  |  | X |  |  |  |  | X |  | X |  |
| Deep Tobacco |  |  | X |  | X |  |  | X |  |  |  | X |  |  |  |  |  | X |  |  | X |
| Curlew |  |  |  |  |  |  |  |  |  |  |  |  |  |  |  |  | X |  | X |  |  |
| Glovers #1 | X |  |  | X |  |  |  | X |  |  |  | X |  |  | X |  |  | X |  |  | X |
| Glovers #2 |  | X | X | X | X |  |  |  | X |  |  | X |  |  | X |  |  |  |  | X | X |
| Glovers #3 |  |  |  |  | X |  |  |  |  |  |  |  |  |  |  |  |  |  |  |  | X |
| Glovers Atoll |  |  |  | X | X | X |  |  | X |  |  |  | X |  |  |  |  |  |  | X |  |
| Patch Reef |  |  |  |  | X | X |  |  |  |  |  |  | X | X |  |  |  | X |  |  |  |
| Raph’s Wall | X |  | X | X | X |  |  | X |  |  |  | X |  |  | X |  |  | X | X | X | X |
| Station |  |  | X |  | X |  |  |  |  |  |  |  |  |  |  |  |  |  |  |  | X |
| SW/CB |  | X | X |  | X |  |  |  | X | X | X | X | X | X | X |  | X | X | X | X | X |
| Tobacco Shallow |  |  |  |  | X |  |  |  | X |  | X | X | X |  | X |  | X | X | X | X |  |
| Wonderland |  |  | X | X | X | X |  |  |  | X |  | X | X | X | X |  | X | X |  | X | X |

Supplementary Table S4: Sites or region where each sponge species was collected (denoted by number of replicates within a cell) within the Caribbean for microbiome analysis. The total number of each sponge species collected across all sites for microbiome analysis is also shown for reference

| Site | *Aiolochroia crassa* | *Amphimedon compressa* | *Aplysina cauliformis* | *Aplysina fulva* | *Callyspongia (Cladochalina) vaginalis* | *Chondrilla caribensis* | *Ectyoplasia ferox* | *Iotrochota birotulata* | *Ircinia campana* | *Ircinia felix* | *Mycale (Mycale) laevis* | *Niphates erecta* | *Verongula rigida* | *Xestospongia muta* |
| --- | --- | --- | --- | --- | --- | --- | --- | --- | --- | --- | --- | --- | --- | --- |
| Total N Sampled (Microbiome) | 16 | 27 | 30 | 27 | 19 | 16 | 15 | 27 | 24 | 16 | 16 | 25 | 28 | 8 |
| Saigon Bay | 4 | 4 | 4 | 4 | 4 | 4 |  | 4 | 4 | 4 | 4 | 4 | 4 | 4 |
| Crawl Cay | 4 | 4 | 4 | 4 | 4 | 4 | 4 | 4 | 4 | 4 | 4 | 4 | 4 |  |
| Isla Pastores | 4 | 4 | 4 | 4 |  | 4 | 4 | 3 |  | 4 | 4 |  | 4 |  |
| Honduras North |  | 4 | 6 | 4 | 4 |  |  | 4 | 4 |  |  | 4 | 4 |  |
| Honduras South |  | 4 | 4 | 3 | 4 |  |  | 4 | 4 |  |  | 4 | 4 |  |
| Belize |  | 4 | 4 | 4 | 3 | 3 | 3 | 4 | 4 |  |  | 5 | 4 | 4 |
| Wonderland | 4 | 4 | 4 | 4 |  | 4 | 4 | 4 | 4 | 4 | 4 | 4 | 4 |  |

Supplementary Table S5. GenBank accession numbers of gene sequences used to build a phylogeny of focal sponges.

| **Species** | **18S** | **28S** |
| --- | --- | --- |
| ***Aiolochroia crassa*** | KC901954 | KC869494 |
| ***Amphimedon compressa*** | KC902400 | JN178945 |
| ***Aplysina cauliformis*** | KC902201 | KC869470 |
| ***Aplysina fulva*** | KC902200 | KC869518 |
| ***Callyspongia vaginalis*** | EU863813 | EU863806 |
| ***Chondrilla caribensis*** | KC901951 | KC869604 |
| ***Ectyoplasia ferox*** | EU702415 | KC869540 |
| ***Iotrochota birotulata*** | KC902147 | AY561884 |
| ***Ircinia campana*** | KC902359 | KC869531 |
| ***Ircinia felix*** | KC902297 | -- |
| ***Monanchora arbuscula*** | KC902187 | KC869447 |
| ***Mycale laevis*** | -- | KC869556 |
| ***Niphates erecta*** | KC902280 | JN178944 |
| ***Plakina trilopha*** | HM118546 | HM118560 |
| ***Plakortis halichondrioides*** | HM118543 | KC869492 |
| ***Verongula rigida*** | -- | KC869452 |
| ***Xestospongia muta*** | KC902281 | -- |

| Species | Mean δ^15^N (+/-SE) | Mean δ^13^C (+/-SE) | Mean C:N (+/-SE) |
| --- | --- | --- | --- |
| *Aplysina cauliformis* | 2.12 (0.08) | -19.20 (0.05) | 3.91 (0.01) |
| *Amphimedon compressa* | 4.63 (0.08) | -18.29 (0.18) | 4.60 (0.10) |
| *Aiolochroia crassa* | 3.05 (0.11) | -17.63 (0.12) | 3.78 (0.03) |
| *Aplysina fulva* | 2.19 (0.09) | -19.44 (0.09) | 4.22 (0.10) |
| *Chondrilla caribensis* | 2.28 (0.13) | -17.66 (0.21) | 3.90 (0.06) |
| *Callyspongia vaginalis* | 3.49 (0.10) | -16.73 (0.20) | 3.57 (0.02) |
| *Ectyoplasia ferox* | 2.87 (0.15) | -18.72 (0.14) | 5.35 (0.08) |
| *Iotrochota birotulata* | 3.92 (0.06) | -17.36 (0.14) | 4.16 (0.03) |
| *Ircinia campana* | 1.47 (0.12) | -17.21 (0.12) | 3.85 (0.03) |
| *Ircinia felix* | 1.06 (0.15) | -17.14 (0.13) | 3.79 (0.04) |
| *Monanchora arbuscula* | 4.26 (0.10) | -18.07 (0.15) | 4.05 (0.05) |
| *Mycale laevis* | 2.98 (0.17) | -16.95 (0.13) | 3.92 (0.04) |
| *Niphates erecta* | 3.90 (0.10) | -17.54 (0.15) | 4.17 (0.09) |
| *Verongula rigida* | 3.24 (0.09) | -17.69 (0.11) | 4.00 (0.02) |
| *Xestospongia muta* | 3.92 (0.08) | -20.43 (0.09) | 4.62 (0.05) |

Supplementary Table S6: Mean (+/-SE) isotope (δ^15^N and δ^13^C) and elemental (C:N) data for 15 common Caribbean sponge species collected throughout the Caribbean. Sample sizes match those found in Table S3.

Supplementary Table S7: Mean (+/- standard error) of microbiome diversity metrics (*S*: ASV richness, *H’*: Shannon index, and *D*: inverse Simpson index) for each host species across the Caribbean.

| Species | *S* | *H’* | *D* |
| --- | --- | --- | --- |
| *Aiolochroia crassa* | 216.75 (14.3) | 4.56 (0.1) | 60.45 (3.5) |
| *Amphimedon compressa* | 206.44 (17.2) | 2.20 (0.1) | 3.78 (0.5) |
| *Aplysina cauliformis* | 291.43 (15.8) | 4.68 (0.1) | 62.37 (3.7) |
| *Aplysina fulva* | 287.81 (7.4) | 4.66 (0.1) | 60.47 (3.2) |
| *Callyspongia vaginalis* | 247.37 (36.6) | 1.79 (0.2) | 2.89 (0.5) |
| *Chondrilla caribensis* | 138.06 (12.1) | 3.61 (0.1) | 21.40 (1.4) |
| *Ectyoplasia ferox* | 194.47 (13.2) | 3.80 (0.1) | 27.66 (1.0) |
| *Iotrochota birotulata* | 151.41 (16.1) | 1.07 (0.1) | 1.57 (0.1) |
| *Ircinia campana* | 147.25 (5.3) | 4.20 (0.1) | 43.57 (1.8) |
| *Ircinia felix* | 146.19 (5.1) | 4.08 (0.1) | 39.17 (3.2) |
| *Mycale laevis* | 615.50 (106.3) | 3.22 (0.3) | 11.91 (5.6) |
| *Niphates erecta* | 223.60 (45.5) | 1.88 (0.2) | 5.49 (2.2) |
| *Verongula rigida* | 265.79 (5.1) | 4.69 (0.1) | 62.07 (2.6) |
| *Xestospongia muta* | 248.88 (14.8) | 4.21 (0.2) | 29.87 (5.9) |

Supplementary Table S8: Mean (+/- standard deviation) relative abundance of 10 microbial phyla that had a relative abundance of at least 1% in the microbiomes of host sponges across the Caribbean.

| Phylum | Abundance |
| --- | --- |
| Proteobacteria | 54% ± 23% |
| Chloroflexi | 11% ± 9% |
| Cyanobacteria | 8% ± 6% |
| Acidobacteria | 7% ± 5% |
| PAUC34f | 4% ± 3% |
| Actinobacteria | 3% ± 2% |
| Bacteroidetes | 3% ± 1% |
| Gemmatimonadetes | 3% ± 2% |
| Thaumarchaeota | 2% ± 2% |
| SBR1093 | 2% ± 2% |


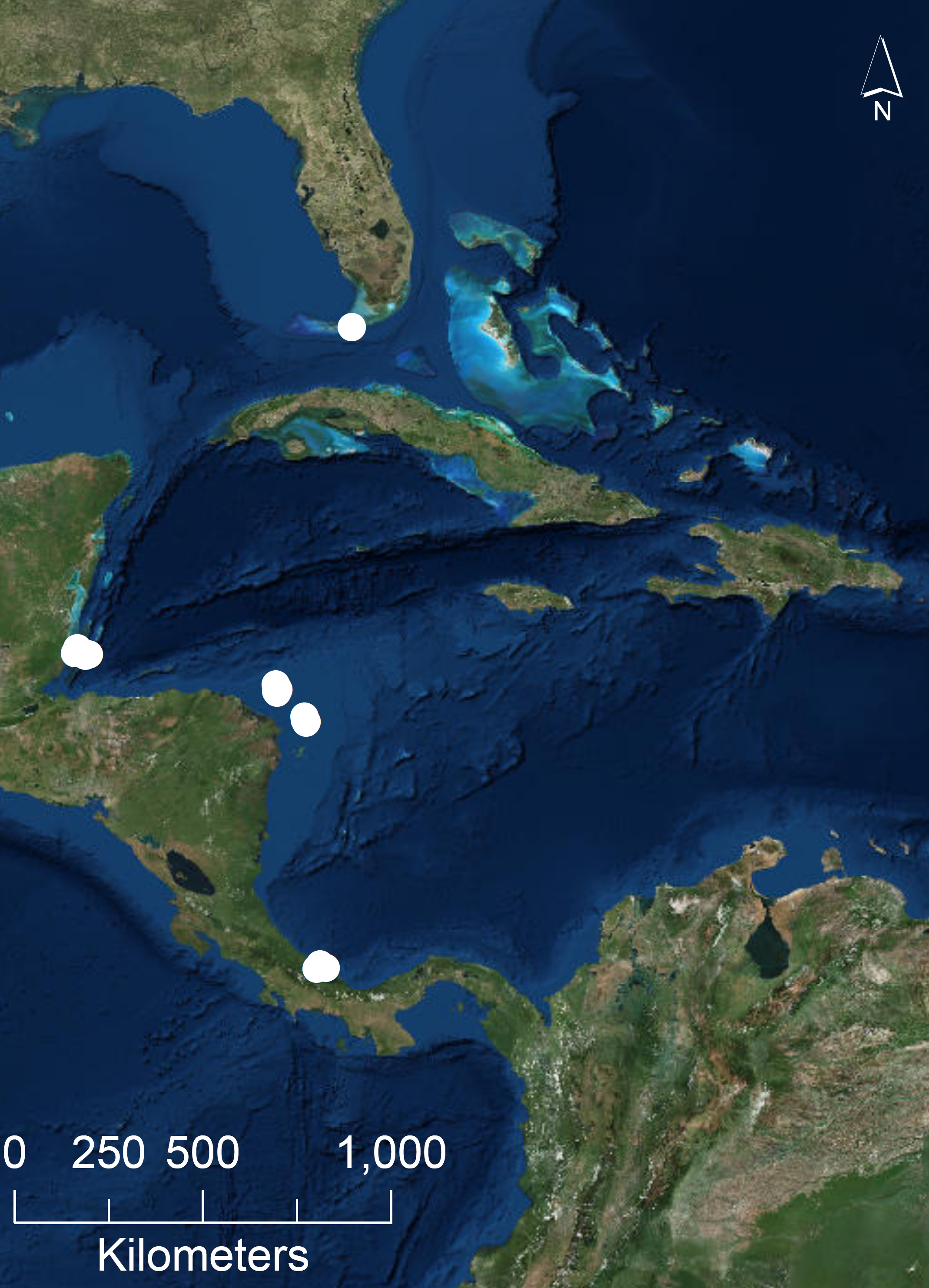


Figure S1: Caribbean-scale map with four geographic regions where sponge sampling took place.


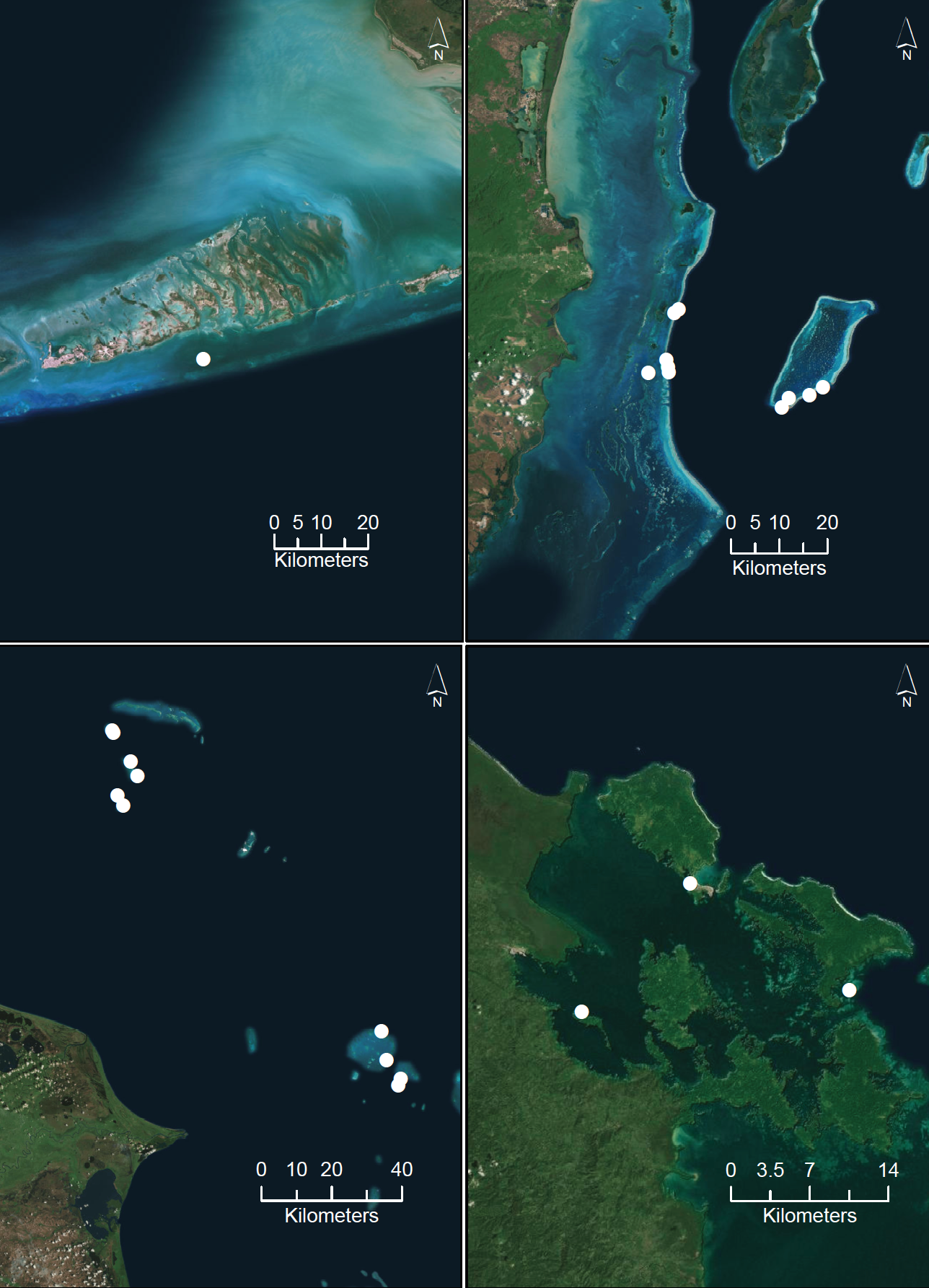


Figure S2: Regional-scale map of sites (white dots) in the Caribbean where sponge collections were carried out. Regions include (clockwise from upper left) the Florida Keys, the Mesoamerican Reef of Belize, the Bocas del Toro archipelago of Panama, and the Miskito Cays of Honduras.


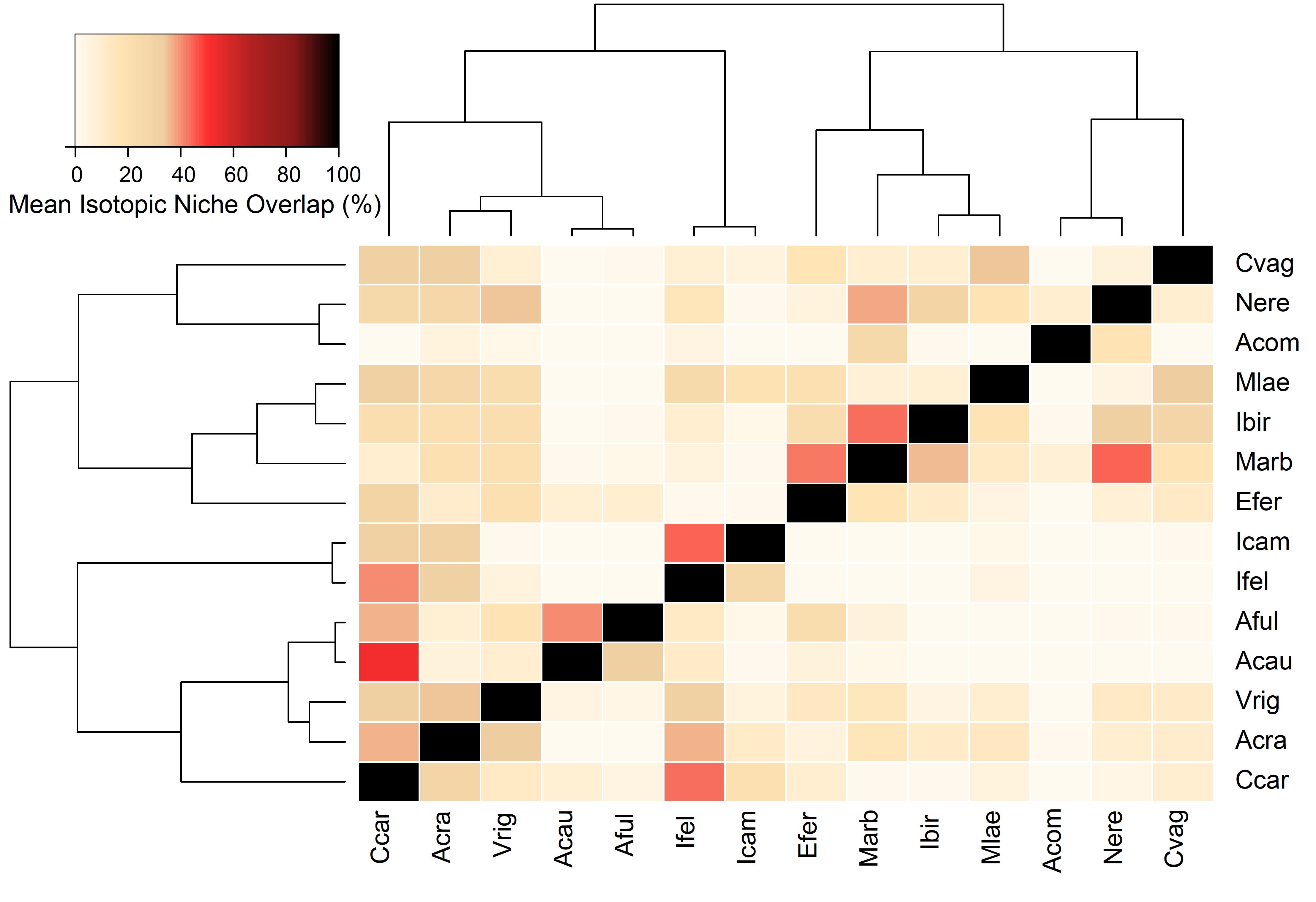


Figure S3: Mean (from eight sites across the Caribbean) pairwise isotopic niche overlap shown via heatmap. Values are directional for each comparison, with cells above the shaded (black) cells representing the overlap of species in columns into the isotopic niche of species in rows; below the black cells represent the overlap of species in rows into the isotopic niche of column species. Host sponges are grouped by host phylogeny.


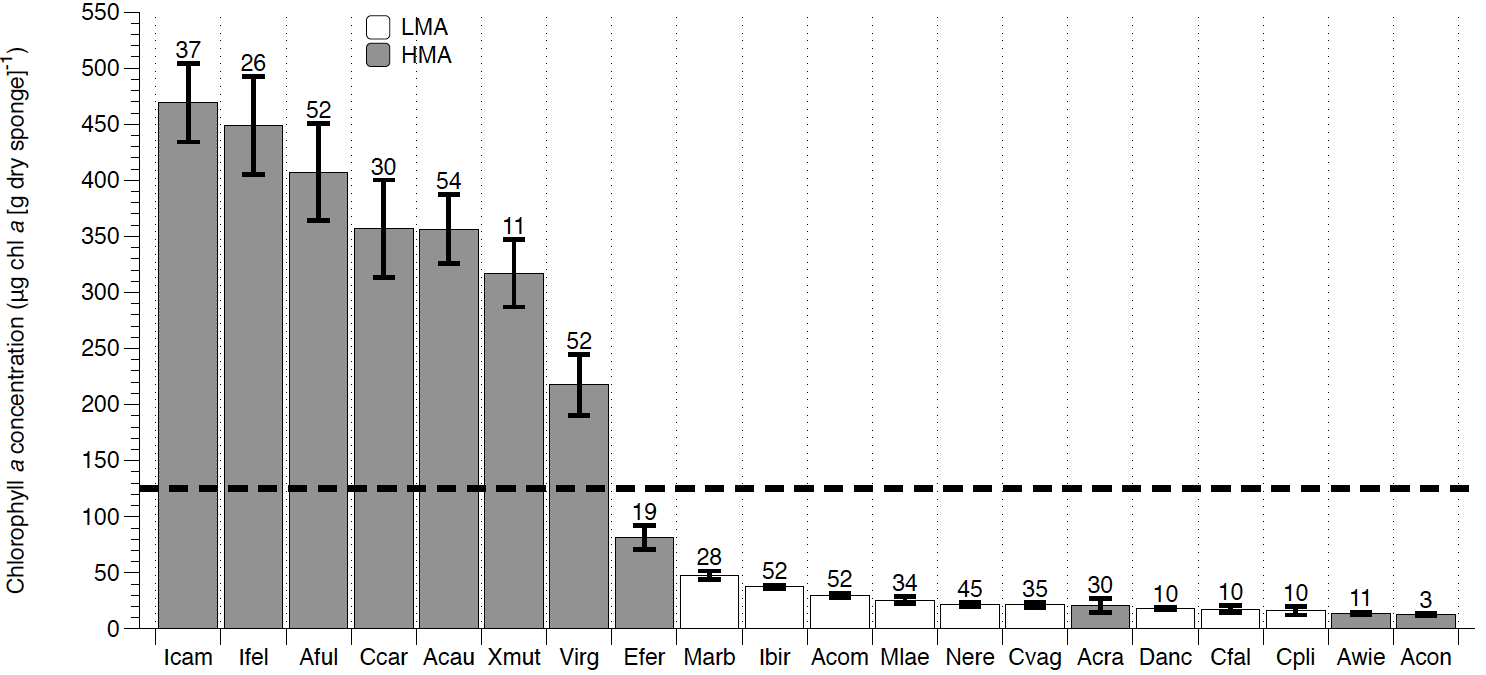


Figure S4: Mean Chlorophyll *a* concentration (+/- SE) of 20 common sponge species collected from sites in Panama, Honduras, and the Florida Keys. The horizontal black line indicates the cutoff above which sponges are considered to have high chlorophyll *a* values (>125 μg chl *a* [g sponge tissue]^-1^; (24)). The overall microbial abundance (High or Low [HMA or LMA]; (2,25,26) of each species is also included for reference. Abbreviations represent the first letter of the genus name, followed by the first three letters of the specific epithet. See Table S1 for full names. Sample sizes are shown above each column for reference.


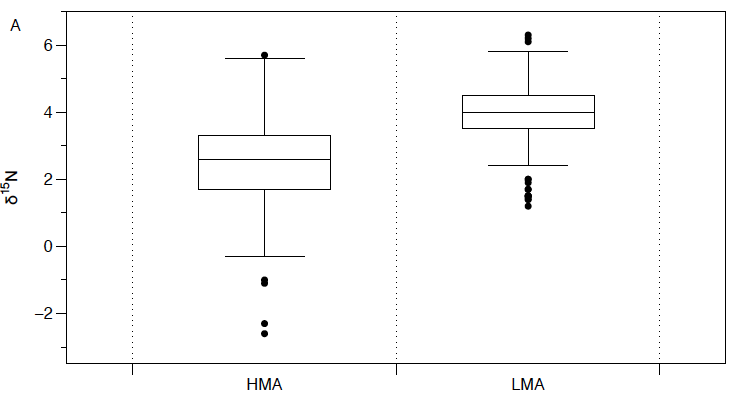


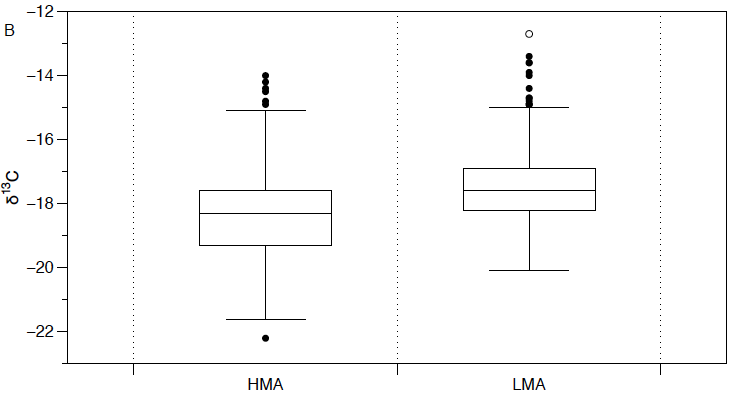


Figure S5: Box and whisker plots representing the mean (horizontal solid line) and range of isotope values δ^15^N (A) and δ^13^C (B) between HMA (High Microbial Abundance) and LMA (Low Microbial Abundance) groups.


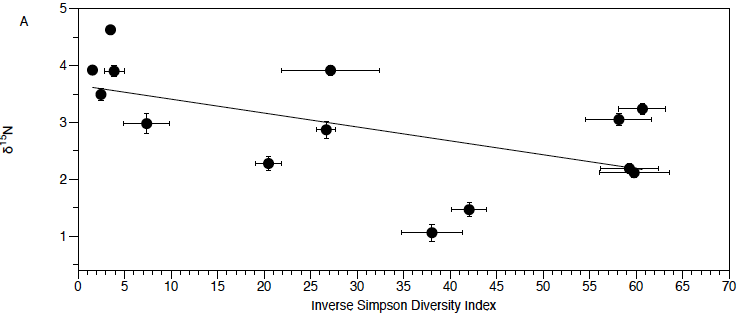


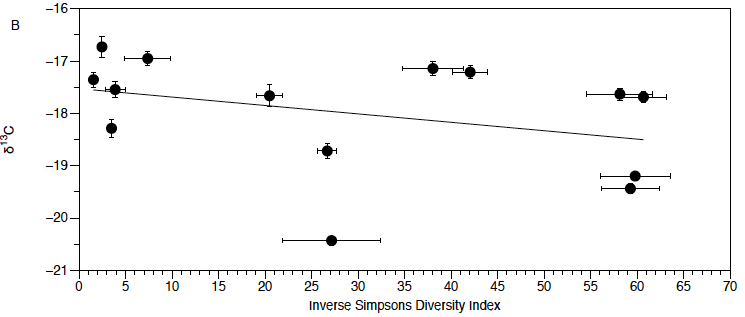


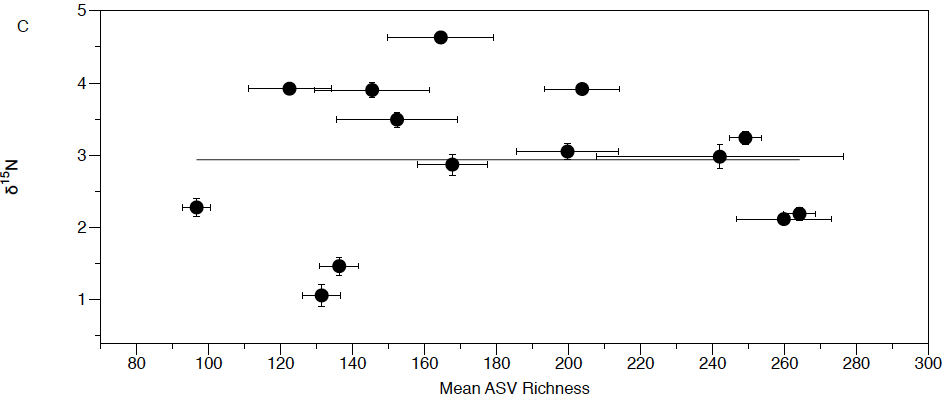


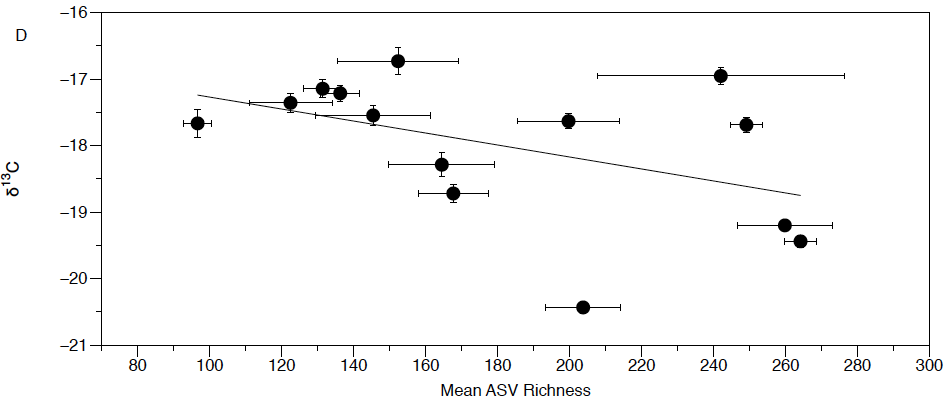


Figure S6: Relationships between mean (+/-SE) isotope values (δ^15^N and δ^13^C) and mean (+/-SE) measures of microbial community diversity (A and B: inverse Simpson’s Index and C and D: ASV richness) in sponge samples from across sites in the Caribbean.


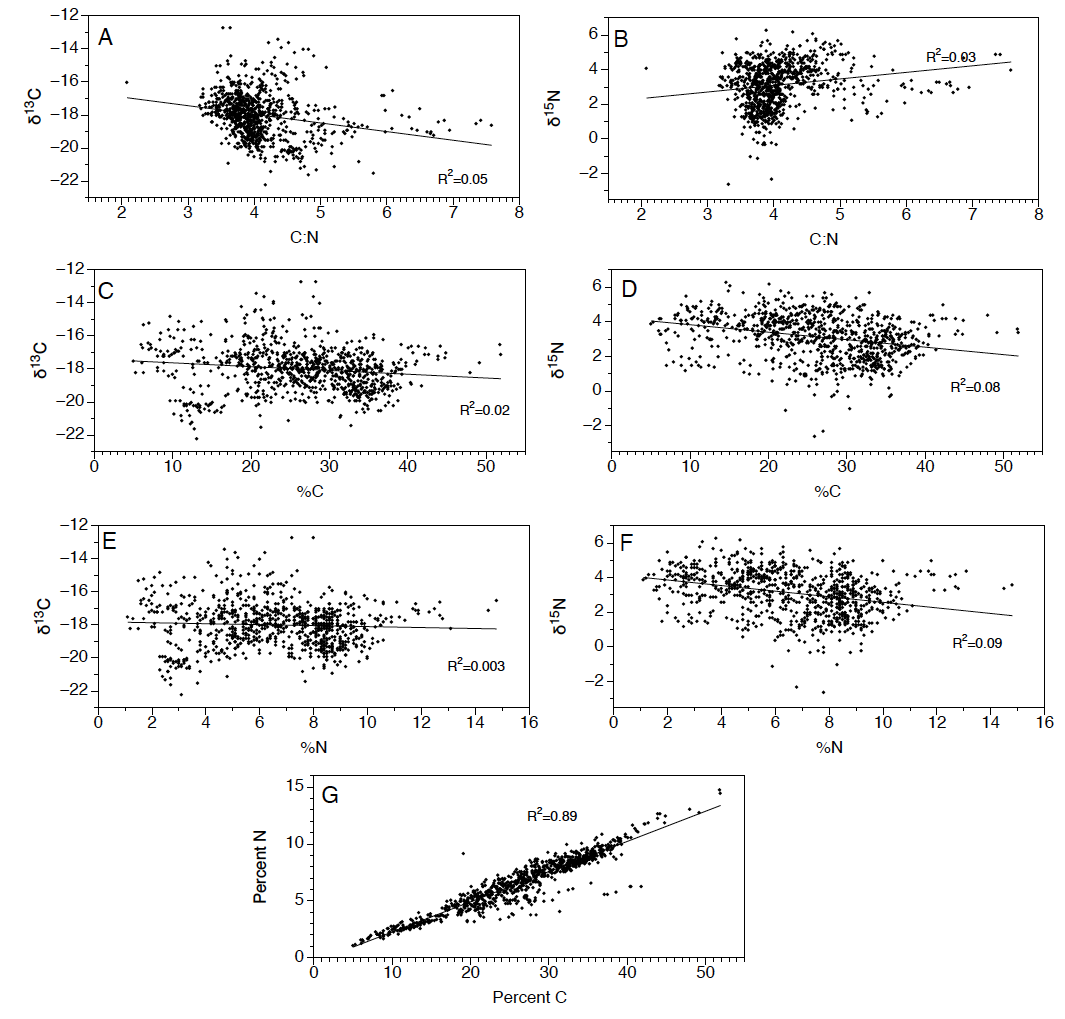


Figure S7: Relationships (via linear regressions) between isotope (δ^15^N and δ^13^C) and elemental (%C, %N, and C:N) values in sponge samples from across sites in the Caribbean. A: δ^13^C vs. C:N; B: δ^15^N vs. C:N; C: δ^13^C vs. %C; D: δ^15^N vs. %C; E: δ^13^C vs. %N; F: δ^15^N vs. %N; and G: %C vs. %N. R^2^ values are shown for reference.
